# The regulatory mechanism of a client kinase controlling its own release from Hsp90 chaperone machinery through phosphorylation

**DOI:** 10.1042/BJ20130963

**Published:** 2013-12-10

**Authors:** Xin-an Lu, Xiaofeng Wang, Wei Zhuo, Lin Jia, Yushan Jiang, Yan Fu, Yongzhang Luo

**Affiliations:** *National Engineering Laboratory for Anti-Tumor Protein Therapeutics, Tsinghua University, Beijing 100084, China; †Beijing Key Laboratory for Protein Therapeutics, Tsinghua University, Beijing 100084, China; ‡Cancer Biology Laboratory, School of Life Sciences, Tsinghua University, Beijing 100084, China

**Keywords:** client, heat-shock protein 90α (Hsp90α), phosphorylation switch, protein kinase Cγ (PKCγ), threonine residue set, 17-AAG, 17-*N*-allylamino-17-demethoxygeldanamycin, Aha1, activator of HSP90 ATPase homologue 1, Cdc37, cell-division cycle 37, GAPDH, glyceraldehyde-3-phosphate dehydrogenase, HA, haemagglutinin, Hsp, heat-shock protein, PKC, protein kinase C, qRT-PCR, quantitative reverse transcription–PCR, WT, wild-type

## Abstract

It is believed that the stability and activity of client proteins are passively regulated by the Hsp90 (heat-shock protein 90) chaperone machinery, which is known to be modulated by its intrinsic ATPase activity, co-chaperones and post-translational modifications. However, it is unclear whether client proteins themselves participate in regulation of the chaperoning process. The present study is the first example to show that a client kinase directly regulates Hsp90 activity, which is a novel level of regulation for the Hsp90 chaperone machinery. First, we prove that PKCγ (protein kinase Cγ) is a client protein of Hsp90α, and, that by interacting with PKCγ, Hsp90α prevents PKCγ degradation and facilitates its cytosol-to-membrane translocation and activation. A threonine residue set, Thr^115^/Thr^425^/Thr^603^, of Hsp90α is specifically phosphorylated by PKCγ, and, more interestingly, this threonine residue set serves as a ‘phosphorylation switch’ for Hsp90α binding or release of PKCγ. Moreover, phosphorylation of Hsp90α by PKCγ decreases the binding affinity of Hsp90α towards ATP and co-chaperones such as Cdc37 (cell-division cycle 37), thereby decreasing its chaperone activity. Further investigation demonstrated that the reciprocal regulation of Hsp90α and PKCγ plays a critical role in cancer cells, and that simultaneous inhibition of PKCγ and Hsp90α synergistically prevents cell migration and promotes apoptosis in cancer cells.

## Abstract 2

The present study is the first example to show that a client directly regulates Hsp90 activity, which is a novel level of regulation for the Hsp90 chaperone machinery.

## INTRODUCTION

Hsp90 (heat-shock protein 90) is one of the most conserved heat-shock proteins and plays an essential role in protection from heat shock [1]. The function of Hsp90, however, extends well beyond heat or stress tolerance [2]. As a critical molecular chaperone, Hsp90 is associated with a wide array of client proteins that require the chaperone function of Hsp90 for their activity and stability [3]. Most of the Hsp90 client proteins are kinases and transcription factors which are at the hubs of signal transduction pathways [4]. Through its chaperone activity, Hsp90 regulates diverse cellular functions and exerts marked effects on cell biology, pathology and evolutionary processes [5,6]. Therefore a comprehensive understanding of the regulatory mechanism of Hsp90 functions will not only shed light on fundamental biological processes, but also provide new avenues for therapeutic interventions.

The chaperone function of Hsp90 is modulated at three major levels, its intrinsic ATPase activity, the association with distinct conformation-specific co-chaperones and post-translational modifications [7]. Although the Hsp90 chaperone machinery can be regulated by these three mechanisms, whether or not there is a fourth mechanism is still unknown. Exhaustive analyses, together with crystal structures of Hsp90, have revealed that ATP binding and hydrolysis lead to a series of conformational rearrangements which trigger the chaperone cycle of Hsp90 [8–10]. Furthermore, conformational changes associated with ATP binding and hydrolysis are accompanied with the binding and release of a distinct set of co-chaperones [11,12]. Previous reports showed that several co-chaperones interact with Hsp90 in a sequential manner to assemble functional chaperone machinery [13,14]. For example, Hsp70 and Hsp90 form a multichaperone complex in which both are connected by a co-chaperone called HOP (Hsp70/90-organizing protein) [15,16]. The connection of and the interplay between the two chaperones are crucial for cell viability [3]. Another important co-chaperone Cdc37 (cell-division cycle 37), which interacts with both protein kinases and Hsp90, and, because it is essential for protein kinase maturation, is therefore known as a ‘kinase co-chaperone’ [17,18]. Cdc37 inhibits Hsp90's ATPase activity and is therefore thought to promote assembly of the misfolded kinase into a multichaperone complex [19,20].

Recent studies have demonstrated that the Hsp90 chaperone machinery can be regulated by post-translational modifications including S-nitrosylation, acetylation and phosphorylation. For example, S-nitrosylation at a conserved cysteine residue (Cys^597^) of Hsp90α affects its ATPase activity and N-terminal dimerization, leading to a decrease in its chaperone activity [21]. Hyperacetylation of Hsp90 by knocking down the deacetylase HDAC6 (histone deacetylase 6) negatively regulates the function of Hsp90 by decreasing its affinity for critical co-chaperones [22]. The regulation of Hsp90 chaperone activity by phosphorylation is more complicated, as Hsp90 has been shown to be phosphorylated at multiple sites [23–25]. For example, phosphorylation of Hsp90α at Tyr^38^ by Wee1 has been shown to regulate multiple aspects of its chaperone function [26]. Phosphorylation of Hsp90α at Thr^90^ by PKA (protein kinase A) not only regulates its chaperone machinery, but also mediates its secretion in cancer cells [24,27]. Another study showed that phosphorylation of yeast Hsp90 at Thr^22^ attenuates its interaction with Aha1 (activator of HSP90 ATPase homologue 1) and Cdc37, which decreases its chaperone activity [28].

Once they have matured through their interactions with Hsp90, how client proteins are then released from the Hsp90 chaperone machinery remains largely unclear. Previous reports have shown that this process is ATP-dependent [29] and can be stimulated by the co-chaperone p23 [30]. Apart from regulation by ATPase and co-chaperones, there are indications that post-translational phosphorylation of Hsp90 stimulates the release of such ‘clients’ as shown for pp60^v−src^ [31]. Recently, it has been shown that tyrosine residue phosphorylation of Cdc37 mediated by Yes kinase disrupts the kinase–Hsp90 complex, and tyrosine phosphorylation of Hsp90 can further release Cdc37 from the chaperone machinery [32]. That study explains a co-chaperone-dependent mechanism for the regulation of the Hsp90–kinase interaction, but does not explain the role of kinase clients in the client-release process.

Since post-translational phosphorylation is known to play an important role in regulating Hsp90 chaperone activity, and a large number of Hsp90 clients are kinases, we hypothesize that kinase clients may modulate the Hsp90 chaperone machinery by phosphorylation at a novel level. We chose PKCγ (protein kinase Cγ), a multifunctional serine/threonine protein kinase reported to interact with Hsp90α [33], as a candidate for the present study. PKCγ is mainly expressed in the central nervous system of healthy people, and is barely detectable in other tissues [34]; however, it has been reported that PKCγ is the major conventional PKC in some cancer cells, especially colon carcinoma cells in which PKCγ is critical for metastasis [35]. We systematically studied reciprocal regulations between Hsp90α and its kinase client PKCγ, and identified a new model for the regulation of Hsp90α chaperone machinery through phosphorylation by its kinase client.

## MATERIALS AND METHODS

### Reagents

Mouse anti-Hsp90α monoclonal antibody for immunoblotting and human recombinant Hsp90α protein were from our laboratory's stock. Mouse anti-Hsp90α monoclonal antibody for immunoprecipitation was purchased from Santa Cruz Biotechnology. The following antibodies were obtained from commercial sources: rabbit anti-phospho-serine-PKC substrate, mouse anti-phospho-Thr^514^-PKCγ and mouse anti-phospho-threonine antibodies were from Cell Signaling Technology; rabbit anti-PKCγ, rabbit anti-Cdc37 and mouse anti-Hsp70 antibodies were from Santa Cruz Biotechnology; mouse anti-HA (haemagglutinin) and anti-Myc monoclonal antibodies and anti-HA affinity matrix were from Roche Applied Biosciences; mouse anti-tGFP (turboGFP) monoclonal antibody was from OriGene; and mouse anti-GAPDH (glyceraldehyde-3-phosphate dehydrogenase) monoclonal antibody, rabbit phospho-serine polyclonal antibody, and horseradish peroxidase-conjugated goat anti-mouse and goat anti-rabbit antibodies were from Abmart.

Other reagents were purchased from commercial sources: Protein A/G–agarose and protease and phosphatase inhibitors (Complete™ Protease Inhibitor Cocktail tablets and PhosSTOP phosphatase Inhibitor Cocktail tablets) were from Roche Applied Science; ATP–agarose was from Innova Biosciences; Staurosporine was from Merck; and 17-AAG (17-*N*-allylamino-17-demethoxygeldanamycin) was from Invivogen.

### Cell culture

HeLa and HCT116 cells (A.T.C.C., Manassas, VA, U.S.A.) were cultured at 37°C with an atmosphere of 95% air and 5% CO_2_ in DMEM (Dulbecco's modified Eagle's medium; Wisent) supplemented with 10% FBS (Wisent), 100 units/ml penicillin (Sigma–Aldrich) and 100 μg/ml streptomycin (Sigma–Aldrich).

### Cell transwell assay

The migration efficiency of cells was assessed using 8-μm-pore Transwell filter membrane (Millipore) as described previously [36]. Migrated cells were quantified by counting in eight random fields under an Olympus IX71 optical microscope. Experiments were conducted in triplicate and repeated twice.

### Plasmid and siRNA transfection

HeLa cells were plated into 6-well or 6-cm plates 24 h before plasmid transfection at a confluence of 60–80%. For single transient transfection in 6-well plates, 2 μg of plasmid/well was used with 3 μl of TurboFect™ *in vitro* transfection reagent (Fermentas). For co-transfection, 3 μg of plasmid (1.5 μg each) was used per well with 5 μl of TurboFect™ *in vitro* transfection reagent. Both the plasmid and transfection reagent were doubled in the 6-cm plates. Culture media were refreshed 5–7 h after transfection and cells were harvested for analysis 36–48 h post-transfection.

For siRNA transfection, HeLa cells were transfected using Lipofectamine™ 2000 (Invitrogen). siRNA against human PKCγ and control scrambled siRNA were from Santa Cruz Biotechnology. siRNA against human Hsp70, Hsp90α and Cdc37 were synthesized by GenePharma. Cells were harvested for analysis at 48–72 h post-transfection.

### Cell lysis and immunoprecipitation

Cell lysis and immunoprecipitation were performed in a cold-room (4°C). Whole-cell lysates were prepared using cell lysis buffer consisting of 20 mM Tris (pH 7.5 at 25°C), 150 mM NaCl, 1% Nonidet P40 and 1 mM DTT with protease and phosphatase inhibitor cocktails. The lysates were then centrifuged for 10 min at 14000 ***g***, the pellet discarded and the soluble fraction used as the whole-cell lysate for immunoprecipitation.

For immunoprecipitation, the protein concentration was measured using a BCA protein kit (Pierce). Immunoprecipitation was performed in a 1.5-ml Eppendorf tube and 1 μg of antibody/mg of cell lysate was used. Cell lysates was pre-incubated with Protein A/G–agarose to reduce non-specific binding. Antibody was first incubated with the cleared cell lysate for 1 h with gentle rotation, 20 μl of suspended Protein A/G–agarose was added to each tube and incubation with rotation was continued in a cold-room overnight. After incubation the resin was pelleted with a brief centrifugation at less than 1000 ***g*** and the supernatant was discarded. The remaining resin was washed three times with the ice-cold lysis buffer and finally resuspended with reducing SDS/PAGE loading buffer for further analysis. For anti-HA affinity matrix purification, 20 μl of suspended resin was added directly to the cell lysate, incubated overnight and then washed with ice-cold lysis buffer.

### Immunoblotting

Samples from whole-cell lysate or immunoprecipitated resin were mixed with reducing SDS/PAGE loading buffer, boiled at 100°C for 15 min, subjected to SDS/PAGE (10% or 12% gels) and transferred on to a PVDF membrane (Millipore). The membrane was blocked in TBST [20 mM Tris (pH 7.5), 150 mM NaCl and 0.1% Tween 20] plus 5–10% dried non-fat skimmed milk for 30 min at room temperature (20–25°C). The membrane was incubated with the indicated primary antibodies in PBST (PBS with 0.1% Tween 20) and 1% dried non-fat skimmed milk for at least 2 h at room temperature or overnight at 4°C, washed three times with TBST for 5 min each time at room temperature, and then incubated with the corresponding horseradish peroxidase-conjugated secondary antibodies for 60 min at room temperature. Following five washes with TBST, immunoreactive bands were detected by ECL (Pierce).

### *In vitro* PKC phosphorylation assay

The *in vitro* phosphorylation of Hsp90α by PKC was performed in PKC reaction buffer [20 mM Hepes (pH 7.4), 1.67 mM CaCl_2_, 10 mM MgCl_2_ and 1 mM DTT]. Recombinant Hsp90α was incubated with PKC with or without ATP at 30°C for 30–60 min, and then the sample was mixed with reducing SDS/PAGE loading buffer for further analysis.

### ATP-binding assay

The proteins used for these assays were ectopically expressed in HeLa cells and then immunopurified with anti-Myc antibodies. Proteins were first washed three times with cell lysis buffer and eluted with buffer containing 20 mM glycine (pH 2.2). The eluted samples were immediately neutralized with Tris buffer to a pH of 7.5.

For the ATP-binding assay, proteins in Tris buffer [10 mM Mg^2+^ and 100 mM NaCl (pH 7.5)] were incubated with high-affinity ATP–agarose (Innova) in a cold room for 1 h and then washed three times with the same buffer. The pelleted resin was mixed with reducing SDS/PAGE loading buffer for immunoblotting.

### ATPase assay

The proteins used for these assays were ectopically expressed in HeLa cells and then immunopurified with anti-His antibodies. The eluted samples were immediately neutralized with Tris buffer to a pH of 7.5 and the protein concentration was measured using the BCA protein kit.

For the ATPase assay, an ATP calibration curve was first prepared and then proteins of same concentration were incubated with 100 nM ATP in Tris buffer (pH 7.5) at 37°C for 30 min. The remaining level of ATP was then measured from the supernatant fluid with an ATP bioluminescent kit (FLAA, Sigma–Aldrich) according to the manufacturer's instructions. Experiments were conducted in triplicate.

### cDNA cloning, expression vectors and mutagenesis

cDNAs encoding the entire ORFs of human Hsp90α, Cdc37 and PKCγ were amplified by PCR from a human liver cDNA library. The amplified cDNAs were subcloned into different expression vectors. Briefly, Hsp90α was subcloned into pcDNA3.1/Myc-His with Myc and His epitopes at their N- or C-terminus as described previously [24]. Other proteins were subcloned into pHM6 with an HA epitope at their N-terminus. Mutagenesis was performed using a QuikChange® site-directed mutagenesis kit (Stratagene). All constructs were confirmed by sequencing (Invitrogen).

### LC-MS

Gel slices containing protein bands of interest were excised and digested by sequencing grade modified Trypsin (Promega). The peptide mixture was analysed by LC-MS (Agilent 6300 Series Ion Trap LC, Mass Systems). MS data were extracted and searched against the Swiss-Prot database using ProteinPilot software as described previously [37].

### qRT-PCR

HeLa cells were lysed and total RNA was collected by extraction with TRIzol® (Invitrogen) as described previously [38]. Poly(A) mRNA was reverse-transcribed using the RevertAid First Strand cDNA Synthesis kit (Fermentas) and aliquots of cDNA (1 μl) were used as a template for qRT-PCR (quantitative reverse transcription–PCR).

qRT-PCR was performed using the Light Cycler 480 SYBR Green Master Mix I from Roche Applied Science according to the manufacturer's protocol. The thermal cycler conditions were as follows: pre-incubation for 5 min at 95°C, followed by 40 cycles of amplification at 95°C for 10 s, 55°C for 15 s and 72°C for 8 s. For each qRT-PCR we obtained the slope value and linear range of the standard curve of serial dilutions. All reactions were performed in triplicate. *GAPDH* mRNA levels, a metabolic enzyme whose transcription is not regulated by ER (endoplasmic reticulum) stress, served as an internal normalization standard.

### Separation of the cytosol and membrane fractions

HeLa cells were homogenized on ice in buffer A [25 mM Hepes, 250 mM sucrose (pH 7.4), and protease and phosphatase inhibitor cocktails]. Nuclei were pelleted by centrifugation at 1500 ***g*** for 10 min and organelle components were removed by spinning at 8000 ***g*** for 10 min. The membrane pellet was then obtained by ultracentrifugation at 150000 ***g*** for 1 h using a TLA-120 rotor (Beckman Coulter) and the supernatant was in the cytosol fraction. Membrane pellets were dissolved in buffer B [50 mM Tris (pH 7.5), 1% Nonidet P40, 150 mM NaCl, and protease and phosphatase inhibitor cocktails].

## RESULTS

### PKCγ and Hsp90α interact with each other

**Figure 1:**
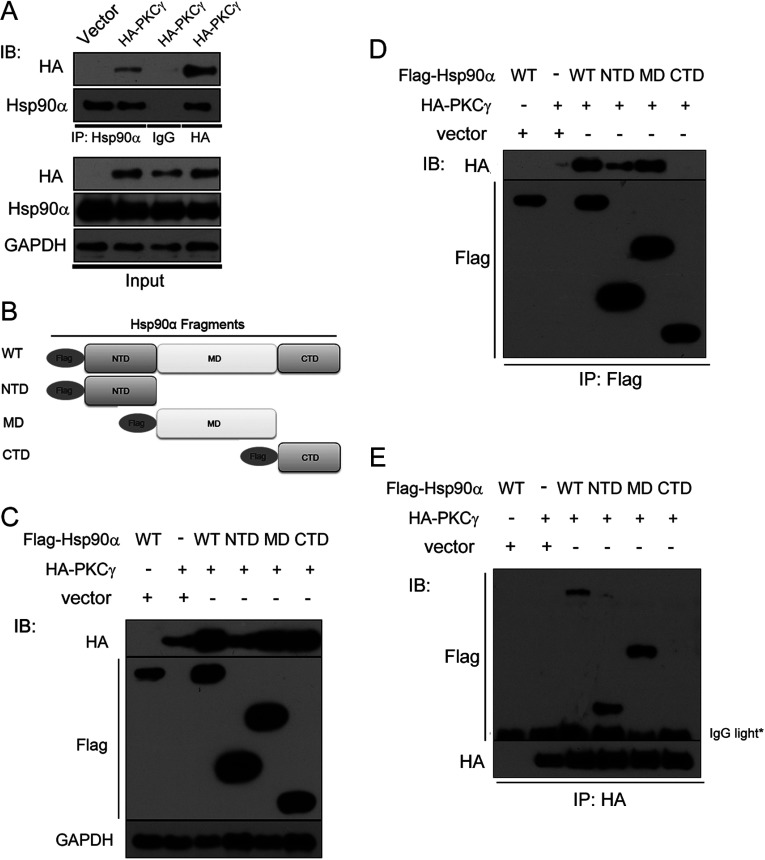
Hsp90α interactions with PKCγ (**A**) Lysates of HeLa cells transfected with control (Vector) or HA-tagged PKCγ-expressing vectors were subjected to immunoprecipitation (IP) with an anti-Hsp90α antibody (IP: Hsp90α), a control IgG (IgG) and an anti-HA antibody (HA). The immunoprecipitates were resolved by SDS/PAGE and immunoblotted with the respective antibodies. (**B**) Schematic diagram of Hsp90α fragments with a FLAG tag. (**C**) The expression level of FLAG-tagged Hsp90α fragments and HA-tagged PKCγ was detected after co-transfection. (**D** and **E**) The interaction of the WT N-terminal domain and middle domains of Hsp90α with PKCγ. Immunoprecipitates were immunoblotted with an anti-FLAG antibody to detect the immunoprecipitation efficiency and with an anti-HA antibody to detect the interacting domain of Hsp90α with PKCγ (**D**) and vice versa (**E**). CTD, C-terminal domain; IB, immunoblotting; MD, middle domain; NTD, N-terminal domain.

To determine whether PKCγ is chaperoned by Hsp90α, first, we confirmed the previously reported physical interaction between Hsp90α and PKCγ. HeLa cells were transfected with a plasmid expressing HA-tagged PKCγ. After whole cells were lysed and immunoprecipitated with a monoclonal anti-Hsp90α antibody, the immunoprecipitates were then analysed by Western blotting with an anti-HA antibody. We found that PKCγ was co-immunoprecipitated with Hsp90α (Figure 1A). Moreover, Hsp90α was consistently co-precipitated with HA-tagged PKCγ immunoprecipitated by the anti-HA antibody (Figure 1A).

Next we mapped the region of Hsp90α that mediates its interaction with PKCγ. Studies have reported that a conserved motif in the C-terminal domain of PKC mediates its interaction with Hsp90 [33], whereas which region of Hsp90α binds PKCγ is still unknown. Hsp90α exists mainly as a flexible homodimer in mammalian cells and each monomer contains three domains: an N-terminal ATP-binding domain, a middle domain and a C-terminal dimerization domain [39]. We constructed transfection vectors encoding FLAG-tagged WT (wild-type) Hsp90α and three truncations including the N-terminal domain, the middle domain and the C-terminal domain (Figure 1B). HeLa cells were then co-transfected with FLAG-tagged Hsp90α truncations and HA-tagged PKCγ. After confirming the expression of FLAG–Hsp90α fragments and HA–PKCγ by Western blotting (Figure 1C), we then analysed the interactions between Hsp90α fragments and PKCγ by a co-immunoprecipitation assay. Our results showed that both the N-terminal and middle domains were co-precipitated by PKCγ and vice versa (Figures 1D and 1E). Taken together, these results demonstrate that Hsp90α interacts with PKCγ through its N-terminal and middle domains.

### PKCγ is chaperoned by Hsp90α which thus activates its kinase activity

PKCγ belongs to the conventional subfamily of PKC, which needs to be transferred from the cytosol to the plasma membrane for activation [40]. After confirming that Hsp90α interacts with PKCγ, we wondered whether Hsp90α also stabilizes PKCγ and facilitates its cell membrane translocation and activation. We focused on Thr^514^ because it is a major phosphorylation site in PKCγ and, moreover, its phosphorylation status is an indicator of PKCγ kinase activation [41].

**Figure 2:**
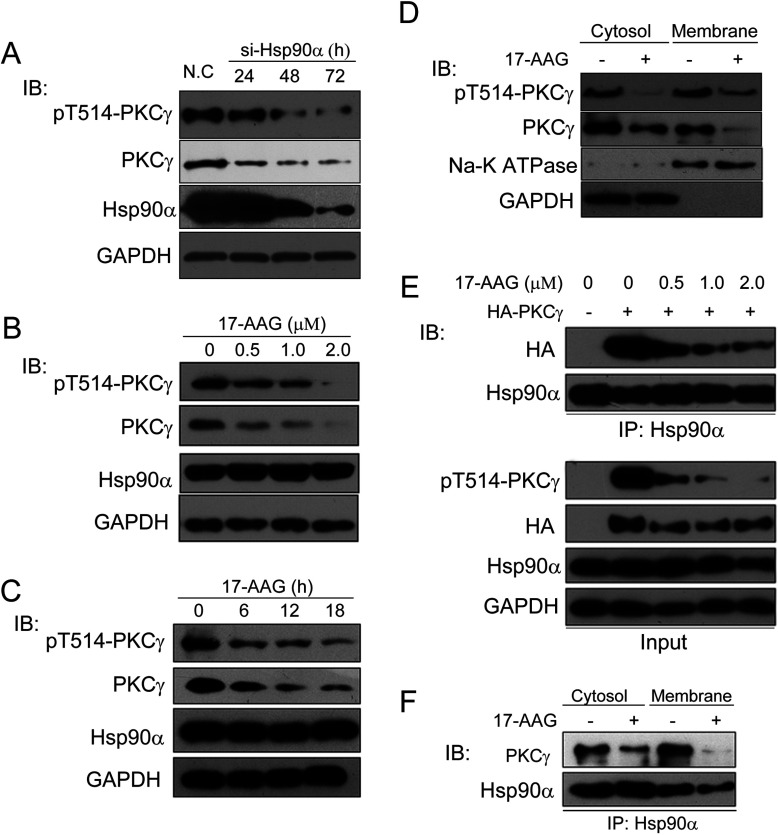
PKCγ chaperoning by Hsp90α (**A**) Whole-cell lysates were prepared from control siRNA or Hsp90α siRNA (si-Hsp90α)-transfected HeLa cells. Protein levels of Hsp90α and PKCγ and the phospho-Thr^514^ level of PKCγ were then detected by their respective antibodies. N.C, negative control. (**B**) HeLa cells treated by 17-AAG in a dose-dependent manner for 12 h were prepared for SDS/PAGE and protein levels of phopho-Thr^514^-PKCγ, PKCγ, Hsp90α and the loading control GAPDH were then detected by immunoblotting. (**C**) HeLa cells treated by 1 μM 17-AAG for different times were prepared for SDS/PAGE, and then protein levels of phospho-Thr^514^-PKCγ, PKCγ, Hsp90α and the loading control GAPDH were detected by immunoblotting. (**D**) Protein levels of phospho-Thr^514^-PKCγ and PKCγ in different fractions of HeLa cells treated with 1 μM 17-AAG for 12 h were detected by Western blotting. GAPDH and Na^+^/K^+^-ATPase were used as loading controls for the cytosol and membrane fractions respectively. (**E**) HeLa cells were transfected with the control vector or HA–PKCγ, treated with 17-AAG at different concentrations for 12 h and then whole-cell lysates were immunoprecipitated (IP) with an anti-Hsp90α antibody. Co-immunoprecipitated exogenous HA-tagged PKCγ was detected by immunoblotting. (**F**) Hsp90α was immunoprecipitated with an anti-Hsp90α antibody from the cytosol and membrane compartments with/without 17-AAG treatment and then co-precipitated endogenous PKCγ was detected by immunoblotting. IB, immunoblotting.

To test whether the stability and activation status of PKCγ depends upon its interaction with Hsp90α, we knocked down Hsp90α expression in HeLa cells by siRNA and found that the abundance of overall PKCγ protein levels, as well as that of phospho-Thr^514^, were strikingly reduced compared with the controls (Figure 2A). We then confirmed these results by using a pharmacological inhibitor of Hsp90, 17-AAG, a derivative of geldanamycin [42]. After treating HeLa cells with 17-AAG, we measured the mRNA levels of *PKCγ* using qRT-PCR (Supplementary Figure S1 at http://www.biochemj.org/bj/457/bj4570171add.htm). Our data showed that the mRNA level of *PKCγ* was unchanged suggesting that the transcription level of PKCγ was not directly influenced by 17-AAG treatment. We then assayed the protein level and Thr^514^ phosphorylation status of PKCγ by Western blotting after 17-AAG treatment. We found the protein level of PKCγ and its phosphorylation at Thr^514^ in whole HeLa cell lysates were decreased in a time- and dose-dependent manner compared with the controls (Figures 2B and 2C). We then compared the effect of 17-AAG on PKCγ abundance in the membrane compared with the cytosol cell fractions. As shown in Figure 2(D), the PKCγ protein abundance was far more reduced in the membrane fraction than that in the cytosol fraction in 17-AAG-treated cells compared with the controls, indicating that the chaperone activity of Hsp90 is also very critical for PKCγ membrane translocation.

We also found the protein level of membrane Hsp90α was consistent with the inhibition of PKCγ activation induced by its translocation blockade (Figure 2D). To test further that the chaperone function of Hsp90α is critical for the stabilization and activation of PKCγ, HeLa cells were transfected with HA-tagged PKCγ and then treated with 17-AAG at different doses. A co-immunoprecipitation assay revealed that the interaction of Hsp90α and PKCγ was increasingly suppressed when 17-AAG concentrations were increased (Figure 2E). Similar results were observed in both the cytosol and the membrane fraction (Figure 2F). These results confirm that Hsp90α indeed chaperones PKCγ through their interactions, thereby preventing its degradation and facilitating its kinase activation.

### Cdc37 is the key co-chaperone for Hsp90α's chaperoning of PKCγ

We next investigated whether the co-chaperone Cdc37 is involved in the Hsp90α chaperoning of PKCγ. The Hsp90-dependent chaperone cycle requires sequential association and dissociation of various co-chaperones to effectively chaperone and release clients [43]. We focused on Cdc37 because it is known to play an important role as a molecular chaperone in stabilizing newly synthesized kinase proteins and in mediating the loading of protein kinases to Hsp90 [19]. First, we used co-immunoprecipitation to test the interactions among Hsp90α, PKCγ, Hsp70 and Cdc37. PKCγ was shown to pull down all of the other three proteins (Supplementary Figure S2A at http://www.biochemj.org/bj/457/bj4570171add.htm), indicating that they form a complex. Hsp70 and Cdc37 did not interact directly with each other, but both of them could interact with Hsp90α and PKCγ (Supplementary Figure S2B), which suggests that only one of the two is the key co-chaperone for the chaperoning process of PKCγ. To test this hypothesis, we knocked down the expression of Hsp70 and Cdc37, individually and simultaneously, by siRNA. As shown in Supplementary Figure S2(C), Cdc37 appears to play a more important role than Hsp70 in stabilizing PKCγ and mediating PKCγ activation. As expected, knocking down the expression of Hsp70 and Cdc37 simultaneously removed completely the protective effect of Hsp90 on the stability and activity of PKCγ. We found that overexpression of Cdc37 in cells with reduced expression of Hsp70 could partially rescue the stabilization and activation of PKCγ, but overexpression of Hsp70 in cells with reduced expression of Cdc37 did not (Supplementary Figure S2D). In addition, the binding affinity between Hsp90α and PKCγ was more significantly decreased in Cdc37-knocked-down cells compared with Hsp70-knocked-down cells (Supplementary Figure S2E). Collectively, these results show that co-chaperone Cdc37 plays a more important role than Hsp70 in recruiting PKCγ to the Hsp90α chaperone machine.

### PKCγ phosphorylates Hsp90α both *in vitro* and *in vivo*

**Figure 3:**
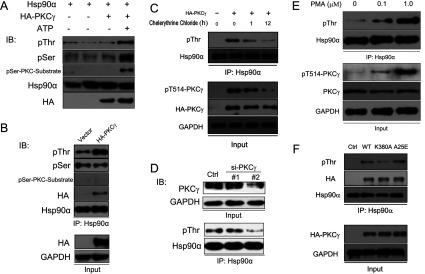
PKCγ phosphorylation of Hsp90α (**A**) Phosphorylation of Hsp90α by PKCγ at threonine and serine residues *in vitro*. pThr, phosphorylation of threonine; pSer, phosphorylation of serine; pSer-PKC-Substrate, phosphorylation of serine sites specifically by PKC. (**B**) Phosphorylation of Hsp90α by PKCγ *in vivo* after exogenous HA–PKCγ transfection into HeLa cells. (**C**) Phosphorylation of Hsp90α was detected upon the treatment with chelerythrine chloride (an inhibitor of PKC). (**D**) The phospho-threonine level of Hsp90α was detected after PKCγ knockdown. Upper panel, the knock-down efficacy was detected by Western blotting. Lower panel, the phospho-threonine level of Hsp90α was probed after immunoprecipitation. (**E**) The phospho-threonine level of Hsp90α was detected upon the treatment with PMA (an agonist of PKCγ). (**F**) The phospho-threonine level of Hsp90α was detected after different forms of PKCγ transfection. K380A, PKCγ with a K380A kinase-dead mutation; A25E, PKCγ with an A25E kinase open mutation; Ctrl, control; IB, immunoblotting; IP, immunoprecipitation.

Once we elucidated that Hsp90α is a critical chaperone of PKCγ and regulates its stabilization and membrane translocation, we asked whether PKCγ can directly phosphorylate Hsp90α. PKCγ was immunoprecipitated from HeLa cells overexpressing HA-tagged PKCγ. After incubation with recombinant Hsp90α, in the presence or absence of ATP, we probed the samples by immunoblotting with antibodies recognizing phospho-threonine residues, phospho-serine residues and a phospho-serine-PKC substrate (which can specifically probe the PKC-mediated phospho-serine residues). We found that both serine and threonine residues of Hsp90α were phosphorylated by PKCγ *in vitro* (Figure 3A); however, only threonine residues were phosphorylated *in vivo* (Figure 3B). We confirmed these results by treating HeLa cells with the PKC inhibitor chelerythrine chloride. Under these conditions, threonine residue phosphorylation of Hsp90α was significantly decreased in time- (Figure 3C) and dose- (Supplementary Figure S3 at http://www.biochemj.org/bj/457/bj4570171add.htm) dependent manners. Having proved that Hsp90α is phosphorylated predominantly on threonine residues, we then showed that threonine phosphorylation of Hsp90α was reduced by knocking down the levels of PKCγ in HeLa cells (Figure 3D). Moreover, when HeLa cells were incubated with PMA, an agonist of PKCγ [44], the threonine residue phosphorylation of Hsp90α was enhanced (Figure 3E). To confirm further the effect of pharmacological inhibition or activation of PKCγ by an independent method, we next examined the ability of two mutated forms of PKCγ, the K380A kinase-dead form and the A25E kinase open form [35], to interact with and phosphorylate Hsp90α in HeLa cells. As shown in Figure 3(F), the interaction between Hsp90α and the three forms of PKCγ was the same, but the phosphorylation levels of Hsp90α transfected with WT-PKCγ was higher than that with K380A-PKCγ, but lower than that with A25E-PKCγ. Taken together, these results demonstrate that PKCγ phosphorylates its chaperone Hsp90α specifically at threonine residues *in vivo*.

### PKCγ phosphorylates a threonine residue set, Thr^115^/Thr^425^/Thr^603^, of Hsp90α

**Figure 4:**
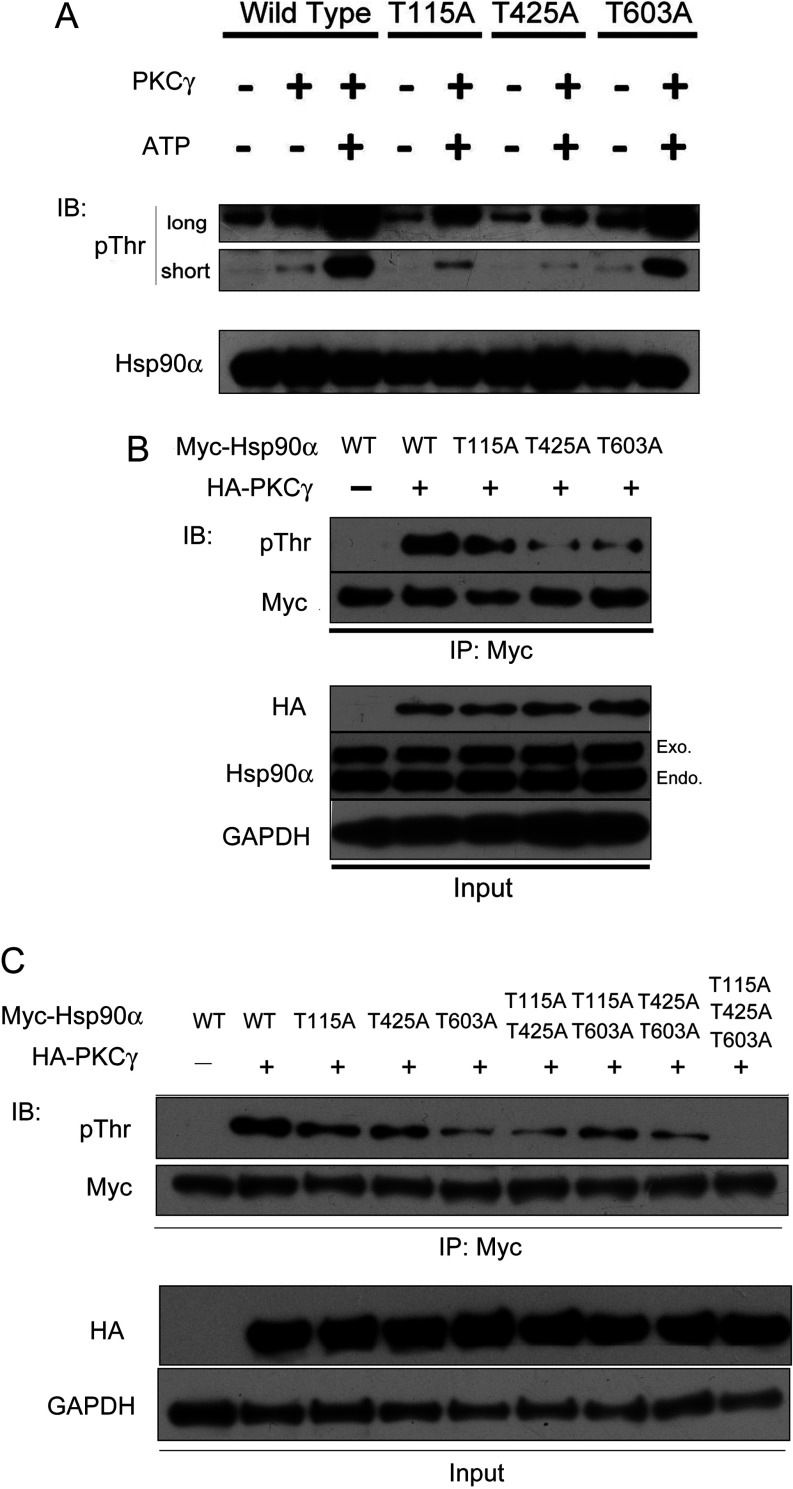
Phosphorylation of Hsp90α Thr^115^, Thr^425^ and Thr^603^ by PKCγ (**A**) Left-hand three lanes, threonine phosphorylation status of WT Hsp90α protein by PKCγ *in vitro*. Right-hand six lanes, threonine phosphorylation status of non-phospho-mimic Hsp90α mutants by PKCγ *in vitro*. (**B**) Threonine phosphorylation status of WT and non-phospho-mimics Hsp90α by PKCγ *in vivo*. HeLa cells transiently expressing Myc-tagged WT, T115A, T425A and T603A Hsp90α and HA-tagged PKCγ were lysed and ectopic Hsp90α proteins were immunoprecipitated with an anti-Myc antibody and immunoblotted with the indicated antibodies. (**C**) HeLa cells transfected with Myc-tagged WT Hsp90α or its non-phospho-mimics (single, double or triple site mutations) and HA-tagged PKCγ were lysed and immunoprecipitated (IP) with anti-Myc antibody and the immunoprecipitates were immunoblotted with an anti-phospho-Thr antibody. IB, immunoblotting; pThr, phosphorylation of threonine.

To identify which threonine residues of Hsp90α are phosphorylated by PKCγ, we purified phosphorylation reaction samples and analysed them by LC-MS. We identified three threonine residues (Thr^115^, Thr^425^ and Thr^603^) as potential phosphorylation candidates (Supplementary Figure S4 at http://www.biochemj.org/bj/457/bj4570171add.htm). To confirm that these sites were phosphorylated by PKCγ, we constructed three Hsp90α non-phospho-mutants (T115A, T425A and T603A), and incubated them with PKCγ *in vitro*. We found that threonine residue phosphorylation in these mutants was attenuated, although the effect in the T603A mutant was not as strong as in the T115A or T425A mutants (Figure 4A). We confirmed this result using an *in vivo* phosphorylation assay (Figure 4B), although the levels of phospho-threonine differed in the *in vitro* compared with the *in vivo* assays.

Hsp90α has a total of 43 threonine residues, so we next queried whether Hsp90α is phosphorylated by PKCγ specifically at the Thr^115^/Thr^425^/Thr^603^ threonine residue set. We found that when all three threonine residues comprising this set were mutated, the threonine phosphorylation of Hsp90α decreased to the basal level (Figure 4C), which strongly suggests that PKCγ phosphorylates Hsp90α only at these three threonine residues. Consequently, we concluded that Thr^115^, Thr^425^ and Thr^603^ are the phosphorylation sites of Hsp90α regulated by PKCγ.

### Threonine residue phosphorylation of Hsp90α by PKCγ affects Hsp90α chaperone machinery

**Figure 5:**
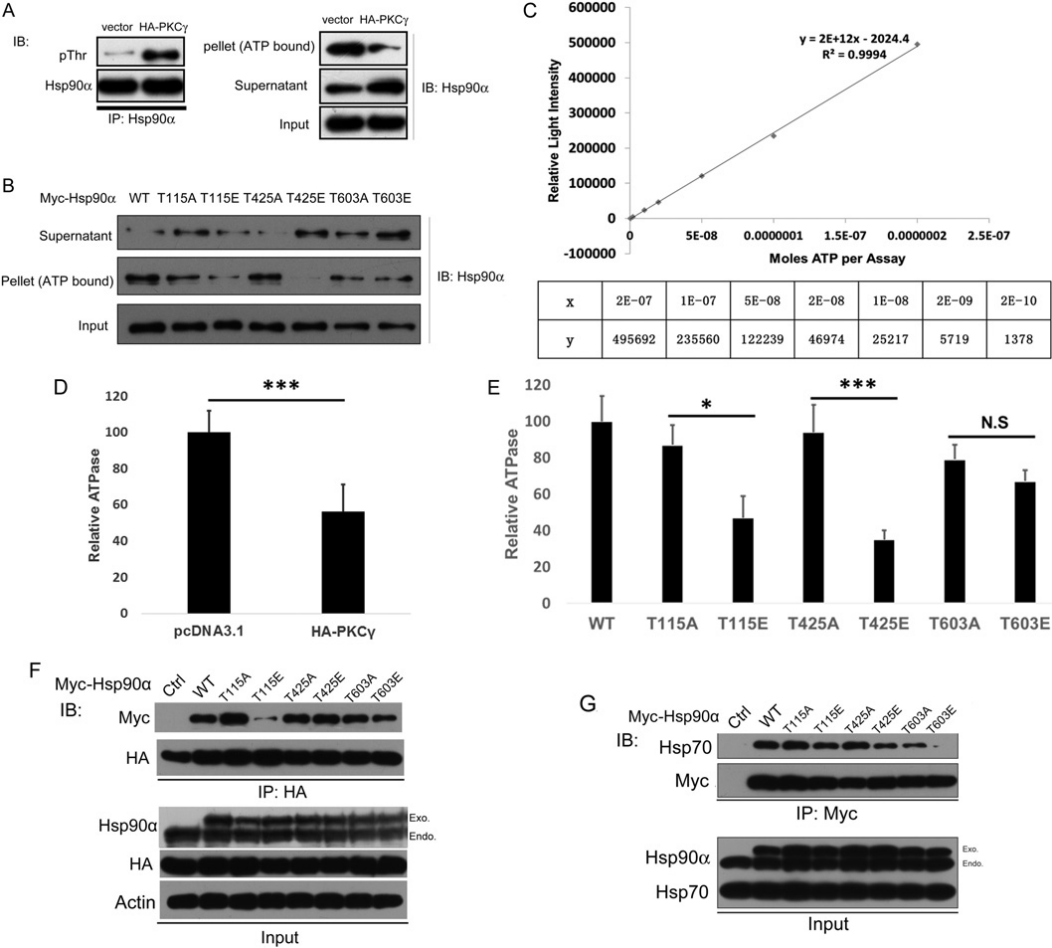
The effect of threonine phosphorylation of Hsp90α by PKCγ on Hsp90α chaperone function (**A**) Binding of two forms of Hsp90α to high affinity ATP–agarose. WT Hsp90α was immunoprecipitated (IP) from control vector-transfected HeLa cells, whereas phospho-Thr-Hsp90α (pThr) was immunoprecipitated from HeLa cells transiently transfected with HA–PKCγ. (**B**) Binding of WT Hsp90α, T115A/E, T425A/E and T603A/E mutants to high affinity ATP–agarose. Proteins were ectopically expressed in HeLa cells and immunoprecipitated with anti-Myc antibodies. Supernatant was from the fraction after incubation with ATP–agarose. The pellet was ATP–agarose resolved with reducing SDS/PAGE loading buffer. (**C**) ATP calibration curve. The *x* axis show moles of ATP per assay and the *y* axis shows the relative light intensity. (**D**) Comparison of the ATPase activities of two endogenous Hsp90α proteins produced from HeLa cells transfected with pcDNA3.1 or HA-PKCγ. ***P<0.001. Results are means±S.D. (**E**) Comparison of ATPase activities of exogenous Hsp90α mutants produced from HeLa cells transfected with the indicated plasmids. ***P<0.001; *P<0.05; N.S, no significant difference. (**F**) HeLa cells co-transfected with control vector (Ctrl), Myc-tagged WT Hsp90α, T115A/E, T425A/E or T603A/E mutants, and HA-tagged Cdc37 were lysed and immunoprecipitated with an anti-Myc antibody. Co-precipitates were then detected by immunoblotting. (**G**) HeLa cells transfected with control vector, WT Hsp90α or T115A/E, T425A/E and T603A/E mutants were lysed and immunoprecipitated with an anti-Myc antibody. Co-precipitated endogenous (Endo) Hsp70 was then detected by immunoblotting. Exo, exogenous; IB, immunoblotting.

We next determined whether Hsp90α's threonine residue phosphorylation by PKCγ could influence the binding affinity of Hsp90α to ATP and its ATPase activity. Endogenous Hsp90α was immunoprecipitated from HeLa cells transfected with control vector or HA–PKCγ. As expected, immunoprecipitated Hsp90α from cells overexpressing PKCγ was highly threonine residue-phosphorylated compared with that from the control cells (Figure 5A, left-hand panel). We then incubated these two forms of Hsp90α with ATP–agarose, in which ATP is attached to agarose beads via its γ-phosphate, and collected the supernatant. The remaining pellet (resin) was extensively washed to eliminate any non-specific binding. Intriguingly, we found that threonine residue phosphorylation of Hsp90α by PKCγ impaired its ATP binding, suggesting that phosphorylation at threonine residues regulates its intrinsic ATPase activity (Figure 5A, right-hand panel). We then used an ATP Bioluminescent Assay kit to detect the relative ATPase activities of the two forms of Hsp90α protein after we prepared the ATP calibration curve (Figure 5C). As shown in Figure 5(D), the ATPase activity of phosphorylated Hsp90α by PKCγ was dramatically decreased.

To confirm further this hypothesis, we tested the ATP-binding affinity and ATPase activity of various Hsp90α phosphorylation mimics or deficient mutants at Thr^115^, Thr^425^ and Thr^603^. The ATPase activity of Hsp90α is located at its N-terminal domain [45] and the middle domain of Hsp90α mediates γ-phosphate interaction, therefore we postulated that Thr^115^ and Thr^425^ phosphorylation would exert a similar effect on the binding affinity of Hsp90α with ATP. Myc-tagged Hsp90α mutants were ectopically expressed in HeLa cells, and proteins were immunoprecipitated with anti-Myc antibodies and then incubated with the ATP–agarose. As shown in Figure 5(B), the phosphorylation-mimic mutants T115E and T425E exhibited decreased ATP-binding affinity compared with the non-phosphorylation mimics T115A and T425A. However, the ATP-binding affinity of the T603E and T603A mutants did not vary significantly. Moreover, the ATPase assay was consistent with the ATP-binding result (Figure 5E). These results indicate that the phosphorylation of Thr^115^ and Thr^425^, rather than Thr^603^, can cause local conformational changes of Hsp90α and subsequently affects its ATP-binding affinity and ATPase activity.

Since lower ATP-binding affinity is a signal of reduced Hsp90α chaperone activity, we speculated that phosphorylation of Hsp90α at these three sites by PKCγ can down-regulate the Hsp90α chaperone machinery. The chaperone activity of Hsp90α requires co-chaperones, therefore we investigated the association of Hsp90α with its co-chaperones after PKCγ phosphorylation by focusing on the binding affinities of the two major co-chaperones Cdc37 and Hsp70. First, we detected the interactions between Hsp90α and Cdc37 by co-immunoprecipitating HA–Cdc37 with WT Myc–Hsp90α or phosphorylation site mutants in transfected HeLa cells. Compared with WT Hsp90α, the phosphorylation-mimic T115E bound with a lower affinity to Cdc37, whereas the non-phosphorylation- and phosphorylation-mimics at Thr^425^ and Thr^603^ bound with similar affinities to Cdc37 (Figure 5F). These results suggest that Thr^115^ phosphorylation decreases kinase client loading to the Hsp90α chaperone machinery by inhibiting the association of Cdc37 with Hsp90α. Hsp70 is another co-chaperone that plays an important role in stabilizing newly synthesized client proteins and transferring them to the Hsp90 chaperone machinery. Therefore we next explored the interactions between these Hsp90α mutants and Hsp70. Using a similar approach to the Cdc37 experiments described above, we found that the interaction between Hsp70 and the Hsp90α T603E mutant was strikingly reduced compared with the controls, whereas Hsp70 interactions with other mutants were only slightly changed (Figure 5G). In summary, these results show that the phosphorylation of Hsp90α by PKCγ reduces Hsp90α's ATP-binding affinity and alters co-chaperone association, and thus down-regulates its chaperone activity.

### Phosphorylation of Hsp90α by PKCγ triggers the release of PKCγ from the Hsp90α chaperone machinery

**Figure 6:**
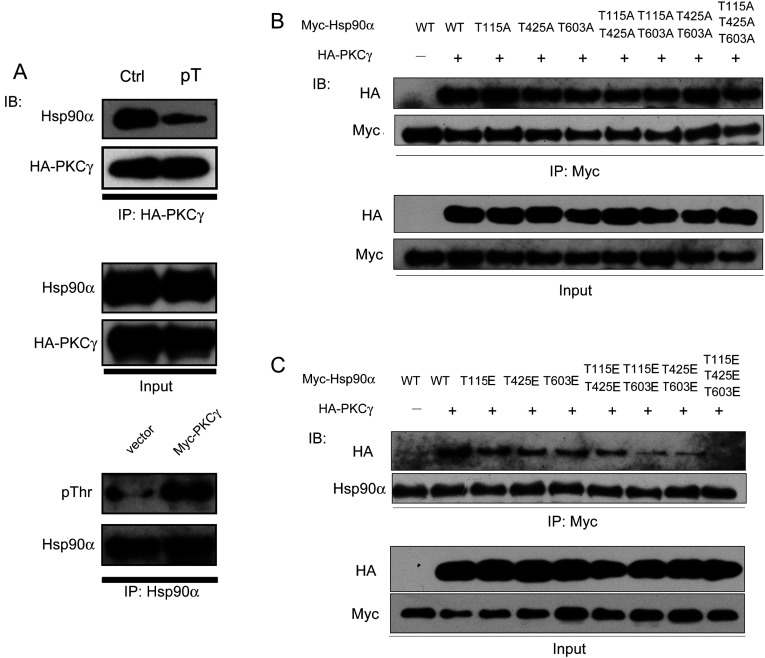
The effects of threonine set phosphorylation of Hsp90α on its interaction with PKCγ (**A**) Physical interaction between the HA–PKCγ protein and two forms of Hsp90α *in vitro*. HA–PKCγ immunoprecipitated (IP) from HeLa cells was transfected with HA–PKCγ. Control Hsp90α (Ctrl) was immunoprecipitated from HeLa cells transfected with a control vector. Phospho-Thr-Hsp90α (pT) was immunoprecipitated from HeLa cells transfected with Myc-tagged PKCγ. (**B** and **C**) HeLa cells co-transfected with HA–PKCγ and Myc-tagged Hsp90α non-phospho (**B**) or phospho (**C**) mutants were lysed and immunoprecipitated with an anti-Myc antibody. The co-precipitated exogenous HA–PKCγ was detected by immunoblotting (IB).

How matured kinase clients are released from the Hsp90 chaperone complex is a question of long-standing interest. Previous studies of the client-release process have mainly focused on the functions of Hsp90's intrinsic ATPase activity and co-chaperones such as Cdc37, Aha1 and PP5 (protein phosphatase 5). We proposed that the phosphorylation of Hsp90α by PKCγ can regulate the disassociation of PKCγ from its Hsp90α chaperone machinery. To prove this hypothesis, we immunoprecipitated WT and threonine residue-phosphorylated Hsp90α from HeLa cells transfected with control vector or overexpressing Myc–PKCγ and incubated these two forms of Hsp90α with the HA–PKCγ protein. As shown in Figure 6(A), the binding affinity between PKCγ and PKCγ-mediated threonine-phosphorylated Hsp90α was strikingly decreased compared with the WT Hsp90α. These data suggest that phosphorylation of Hsp90α, mediated by PKCγ, plays an important role in the disassociation of PKCγ from the Hsp90α chaperone machinery. To confirm further this conclusion, we explored the interactions between PKCγ and the Hsp90α non-phospho- and phospho-mimics. Although all of the Hsp90α non-phosphorylation mimics possessed a similar binding affinity for PKCγ (Figure 6B), the Hsp90α phosphorylation mimics showed increasingly lower binding affinities for PKCγ with an increasing number of mutated sites. Once all of the amino acids in the threonine residue set were converted into phosphorylation mimics, PKCγ completely lost its binding affinity to Hsp90α (Figure 6C). Taken together, these results demonstrate that PKCγ phosphorylation of chaperone Hsp90α triggers its own release from the Hsp90α chaperone machinery.

### Hsp90α regulates PKC-mediated cancer cell migration and survival

**Figure 7:**
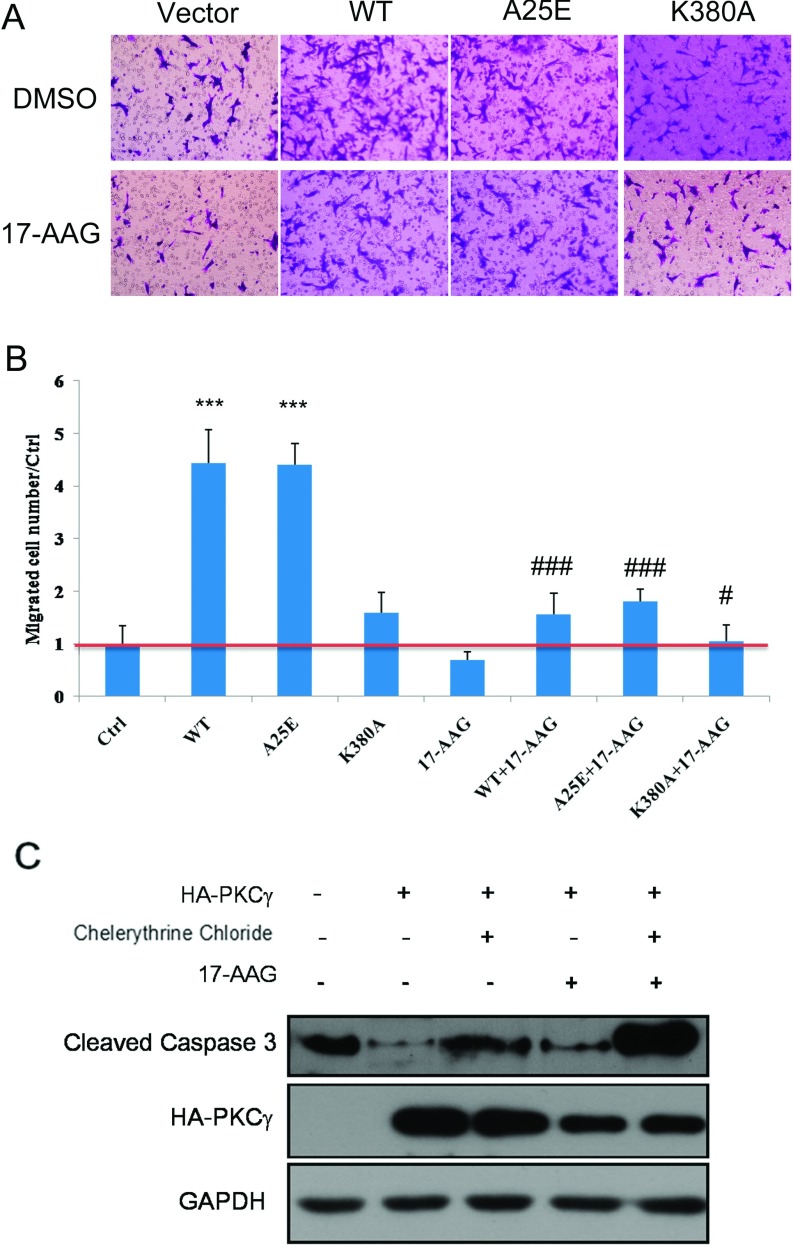
The effects of PKCγ and Hsp90α on cancer cell migration and survival (**A** and **B**) In the cell-migration assay, DMSO or 17-AAG was added to vector-, WT-PKCγ-, A25E-PKCγ- and K380A-PKCγ-transfected groups. After 6 h, migrated cells were examined (**A**) and quantified (**B**) (*n*=8). ***P<0.001; ###*P*<0.001; #*P*<0.05. Results are means±S.D. (**C**) HCT116 cell apoptosis detected by Western blotting of cleaved caspase 3.

Since the interaction of Hsp90α and PKCγ regulates the behaviour of both proteins in living cells, we tested whether Hsp90α also regulates the activity of PKCγ in colon carcinoma cells in which PKCγ is overexpressed. We constructed a kinase-inactive form of PKCγ, K380A-PKCγ, and a constitutively active form, A25E-PKCγ [35]. In a cell transwell assay, HCT116 cells overexpressing WT-PKCγ or A25E-PKCγ showed an increased ability to migrate, compared with the control cells, whereas HCT116 cells overexpressing the kinase-inactive form K380A-PKCγ showed no effect (Figure 7A and quantified in Figure 7B). Moreover, 17-AAG inhibited the ability of PKCγ to promote cell migration, indicating that the chaperone activity of Hsp90α is critical for PKCγ's effect on cell migration (Figure 7A and quantified in Figure 7B). Since PKCγ is known to regulate cell apoptosis, we then tested whether the chaperone activity of Hsp90α is also required for PKCγ's ability to promote cell apoptosis in HCT116 cells by measuring caspase 3 cleavage. As shown in Figure 7(C), overexpression of PKCγ leads to a decrease in cell apoptosis, consistent with an earlier study [35]. Inhibiting the activity of either Hsp90α or PKCγ reversed this phenomenon. Moreover, simultaneously blocking PKCγ/Hsp90α signalling exerted a large synergistic effect.

## DISCUSSION

The present study is the first to investigate the client-protein-modulating Hsp90 chaperone machinery, which not only reveals a novel regulatory mechanism of Hsp90, but also opens up a new direction of chaperone research. Hsp90 is a chaperone for many kinases which play pivotal roles in signal transduction and cellular homoeostasis, and is itself phosphorylated at multiple sites by different kinases. The results of the present study provide new insights to these processes by showing that Hsp90α is directly regulated by a kinase client and how, in turn, the activity of the kinase client is modulated by the Hsp90α chaperone machinery.

**Figure 8:**
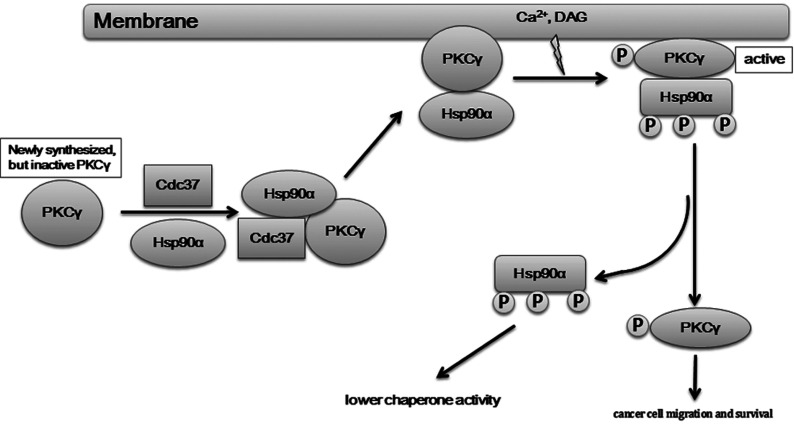
A working model for reciprocal regulations of Hsp90α and PKCγ

The intricate relationship between Hsp90α and its novel kinase client PKCγ can be easily illustrated by an Aesop's fable, *The Farmer and the Viper*. Prior to interacting with its chaperones, newly synthesized PKCγ is like a frozen viper, unstable and inactive. PKCγ then meets the ‘farmer’, Hsp90α, which interacts with PKCγ with the help of the co-chaperone Cdc37. By this interaction, Hsp90α ‘thaws the frozen viper’ and mediates the stabilization and the cytosol-to-membrane translocation of PKCγ. As the ‘revived viper’, PKCγ translocates to the membrane and is further activated by stimulation from secondary messengers, but first, in order to perform its cellular function, PKCγ must disassociate from the Hsp90α chaperone machinery. Activated PKCγ ‘bites the farmer’ by phosphorylating Hsp90α at a specific threonine residue set (Thr^115^/Thr^425^/Thr^603^), which releases it from the Hsp90α chaperone machinery. The overexpressed and activated PKCγ then promotes cancer cell migration and decreases cancer cell apoptosis. However, the phosphorylated Hsp90α (‘bit farmer’) loses all or part of its chaperone activity (Figure 8).

### PKCγ is a client protein of Hsp90α

For a protein to be defined as an Hsp90 client it needs to (i) physically interact with Hsp90 and (ii) inhibition of Hsp90's chaperone function must result in reduced client protein activity. Although it was reported that binding of conventional PKC to the Hsp90α–Cdc37 complex is necessary for its maturation and activation [33], in the present study we present compelling evidence that PKCγ is a client protein of Hsp90α (Figures 1 and 2). PKC, which is a multifunctional cyclic nucleotide-independent serine/threonine protein kinase, mediates a variety of roles including receptor desensitization, modulation of membrane structure, transcriptional regulation, mediation of immune responses, cell growth, and learning and memory [34,46,47]. To regulate these diverse functions, its life cycle must be finely controlled. PKCγ's ability to regulate its own association with Hsp90α suggests a new mechanism by which its activity within the cell is tightly controlled.

### PKCγ phosphorylation of Hsp90α

The results of the present study indicate that, whereas PKCγ phosphorylates Hsp90α only at specific threonine residues *in vivo*, it phosphorylates both serine and threonine residues *in vitro* (Figure 3). This is consistent with studies showing that protein kinase specificity can be determined by its compartmentalization at discrete subcellular locations, from which the protein kinase is recruited to mediate its regulation of substrates [48]. Our data suggests that in the context of Hsp90α regulation, PKCγ activity may be spatially regulated within the cell, such that PKCγ can phosphorylate threonine residues of only a subpool of Hsp90α. However, the mechanistic details of this particular phosphorylation event in the context of all Hsp90α phosphorylation events remains unclear, as does the existence of PKCγ phosphorylation of Hsp90α in other lower eukaryotic or prokaryotic cells. In addition, it is unclear whether the Thr^115^/Thr^425^/Thr^603^ sites of Hsp90α are phosphorylated in a random or sequential manner. Further investigation of these issues will improve our understanding of how Hsp90α post-translational modifications mediate its functions.

### Phosphorylation of Hsp90α by PKCγ decreases its chaperone activity

The results of the present study show that PKCγ-phosphorylated Hsp90α has reduced ATPase activity with a corresponding decrease in chaperone function (Figures 5A and 5B). Interestingly, the Thr^115^ and Thr^425^ phosphorylation sites appear to play a more important role in regulating Hsp90α ATPase activity than the Thr^603^ site. These data are consistent with reports that Thr^115^ resides in the N-terminal domain which mediates the ATPase activity of Hsp90α, whereas Thr^425^ resides in the middle domain which mediates γ-phosphate interaction [8]. In contrast, Thr^603^ is located in the boundary between middle and C-terminal domains, which is distal from the ATPase pocket.

The intact structure of the Hsp90 molecule remains unresolved, probably as a result of its three-domain structure which is joined by what are probably flexible linkers [8]. It is worthwhile to consider the interactions among the three major domains because, by varying its own conformation through such interactions, Hsp90 may mediate its interactions with different co-chaperones and clients and fulfil its extremely diverse biological functions. Hsp90 conformational dynamics are most probably regulated by post-translational modifications. For example, Soroka et al. [49] revealed that Hsp90 is phosphorylated at multiple sites in the middle and C-terminal domains, which permits regulation of the conformational cycle at distinct steps.

We propose a model whereby phosphorylation of the threonine residue set causes a large conformational change affecting all three Hsp90α domains, such that a cascade reaction occurs causing the loss of its chaperone function. In support of such a model, our data shows that phosphorylation of the Thr^115^, Thr^425^ and Thr^603^ sites plays a critical role in mediating the release of matured PKCγ from the Hsp90α chaperone complex. The interactions between Hsp90α and PKCγ are partially reduced when only a single site of the threonine residue set is phosphorylated, and the interaction is abolished when all three sites are phosphorylated (Figure 6C). Whereas partial phosphorylation of these three threonine residues reduces the binding affinities of PKCγ towards Hsp90α, full phosphorylation is required for the release of PKCγ from Hsp90α (Figures 6B and 6C). By comparing phosphorylation of the different combinations of two-site mutations with phospho-mimic groups, we found the interaction of PKCγ and Hsp90α is tighter with the T115E/T425E mutant (Figure 6C) in comparison with the other two combinations. This result suggests that Thr^603^ plays a pivotal role in the client-release process. Given that these three Hsp90α threonine residues are evolutionarily conserved (Supplementary Figure S5 at http://www.biochemj.org/bj/457/bj4570171add.htm), we propose that PKCγ evolved to be phosphorylated at the minimal number of sites to allow the matured PKCγ to ‘escape’ from Hsp90α, such that its release and cellular activities would occur in a controlled manner.

### ‘Phosphorylation switch’ for PKCγ

The Hsp90 chaperone cycle is known to proceed through association and disassociation of both co-chaperones and client proteins [50]. On the basis of the present study, we propose that the Thr^115^/Thr^425^/Thr^603^ threonine residue set serves as a ‘phosphorylation switch’ for Hsp90α: when one or two threonine residues of the threonine set are ‘turned on’ (phosphorylated), Hsp90α loses part of its binding affinity toward PKCγ and when all of the three threonine residues are ‘turned on’, Hsp90α ‘opens’ its doors so that it completely loses its binding affinity toward PKCγ; however, when the threonine set is ‘turned off’ (all dephosphorylated), Hsp90α ‘closes’ its doors and traps PKCγ, that is, Hsp90α has the strongest binding affinity toward PKCγ. In summation, an unphosphorylated threonine set binds PKCγ, whereas a fully phosphorylated form releases PKCγ.

Hsp90 has been reported to be phosphorylated by known client kinases in mammalian cells or in other species such as Src [25], Sch9 (*S. cerevisiae*) [51] etc. Although phosphorylation by these kinase clients can regulate the Hsp90 chaperone machinery, we do not yet know whether such a ‘phosphorylation switch’ exists for these kinase clients and whether non-kinase clients can also actively regulate the Hsp90 chaperone machinery. It will be important for understanding Hsp90–client regulation to resolve these fundamental questions.

### Hsp90α regulates PKCγ-induced cancer cell migration

PKCγ is expressed mainly in neuronal tissues, where it plays roles in neuron development and neuropathic signal transduction. The expression of PKCγ has also been shown to be elevated in some cancer cells, especially colon carcinoma [35]. However, the role of PKCγ in tumour formation and progression is not well understood. In the present study, we demonstrate that overexpression of PKCγ in colon carcinoma cells promotes cell migration and survival (Figure 7), where it is probable that intricate regulation of the Hsp90α and PKCγ interaction is involved.

Taken together, the present study demonstrates a novel regulatory mechanism of the interaction between Hsp90α and its kinase client PKCγ, which provides insights to the regulation of Hsp90α chaperone function by its clients and provides clues to possible therapeutic intervention in PKCγ-elevated cancers.

## Online data

Supplementary data
