# Supplementary material for: The regulatory mechanism of a client kinase controlling its own release from Hsp90 chaperone machinery through phosphorylation
Source: Biochem J. 2013 Dec 10;457(Pt 1):171–83. doi: 10.1042/BJ20130963 (PMC3927929; doi:10.1042/BJ20130963)
Supplement: Supplementary data [file bj4570171add.pdf]

BJ Cell

Biochem. J. (2014) **457**, 171–183 (Printed in Great Britain) doi:10.1042/BJ20130963

online data

SUPPLEMENTARY ONLINE DATA

The regulatory mechanism of a client kinase controlling its own release from Hsp90 chaperone machinery through phosphorylation

Xin-an LU*†‡, Xiaofeng WANG*†‡, Wei ZHUO*†‡, Lin JIA*†‡, Yushan JIANG*†‡, Yan FU*†‡ and Yongzhang LUO*†‡[1]

*National Engineering Laboratory for Anti-Tumor Protein Therapeutics, Tsinghua University, Beijing 100084, China
†Beijing Key Laboratory for Protein Therapeutics, Tsinghua University, Beijing 100084, China
‡Cancer Biology Laboratory, School of Life Sciences, Tsinghua University, Beijing 100084, China

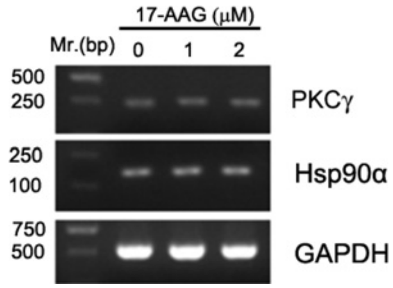

Figure S1 *PKCγ* mRNA level is not changed after 17-AAG treatment

Upper panel, the product of *PKCγ* mRNA qRT-PCR; middle panel, Hsp90α as a positive control; and lower panel, GAPDH as a loading control. Mr. (bp), marker (base pair).

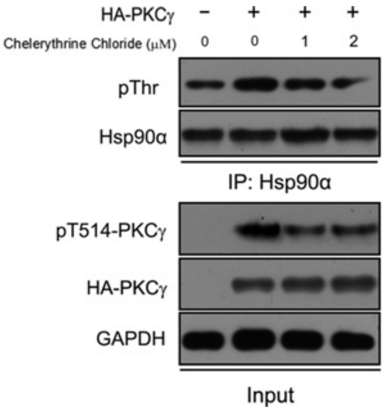

Figure S3 Phosphorylation of Hsp90α was detected upon the treatment with chelerythrine chloride (an inhibitor of PKC) in different doses for 1 h

IP, immunoprecipitation; pThr, phospho-threonine.

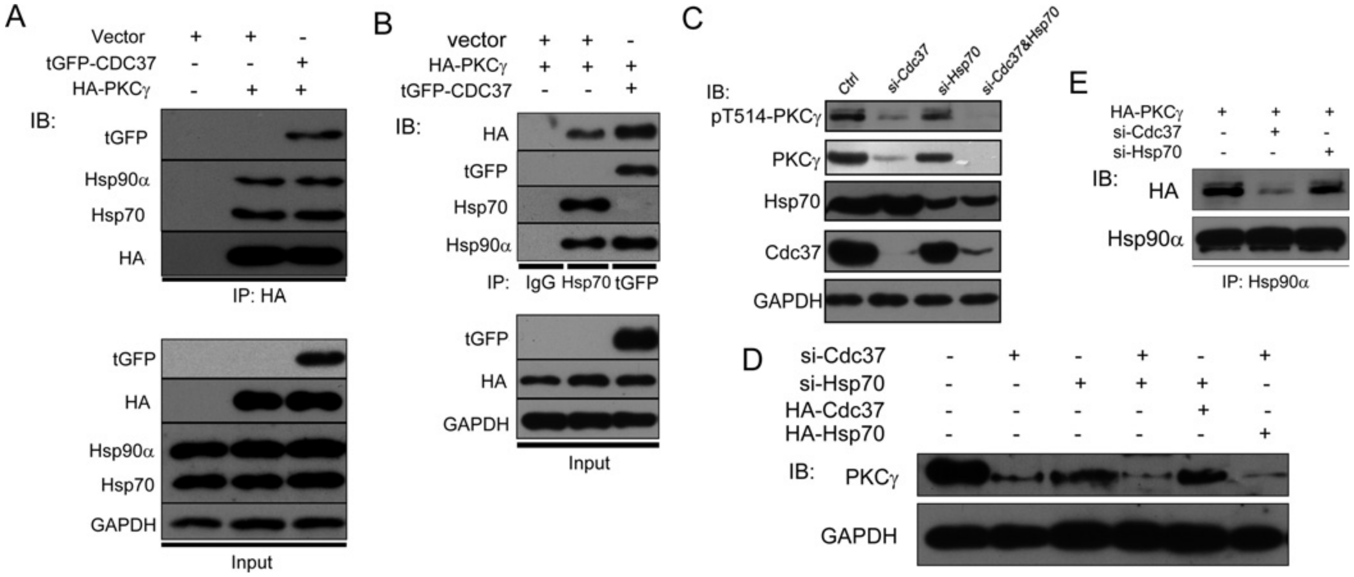

Figure S2 Cdc37 is the core co-chaperone in mediating the chaperoning of PKCγ by Hsp90α

(**A**) HeLa cells transfected with the control vector HA–PKCγ or co-transfected with HA–PKCγ and tGFP (turboGFP)–Cdc37 were lysed and immunoprecipitated using an anti-HA antibody. The co-precipitated endogenous Hsp90α, Hsp70 and exogenous tGFP–Cdc37 were then detected by Western blotting. (**B**) HeLa cells co-transfected with HA–PKCγ and empty vector or with HA-PKCγ and tGFP–Cdc37 were lysed and immunoprecipitated by control IgG, anti-Hsp70 and anti-tGFP antibodies. The co-immunoprecipitates were then detected by immunoblotting. (**C**) Whole-cell lysates were prepared 48 h after si-Cdc37, si-Hsp70 or si-Cdc37 and si-Hsp70 transfection. The protein level of PKCγ and the level of phopho-Thr^{514}-PKCγ were detected. Ctrl, control. (**D**) The endogenous protein levels of PKCγ was detected by immunoblotting after siRNA or overexpressing plasmid transfection. (**E**) The interaction between Hsp90α and HA–PKCγ was detected by co-immunoprecipitation after overexpressing plasmid and siRNA co-transfection. IB, immunoblotting; IP, immunoprecipitation.

[1] To whom correspondence should be addressed (email yluo@tsinghua.edu.cn).

© 2014 The Author(s)
The author(s) has paid for this article to be freely available under the terms of the Creative Commons Attribution Licence (CC-BY) (http://creativecommons.org/licenses/by/3.0/) which permits unrestricted use, distribution and reproduction in any medium, provided the original work is properly cited.

Biochemical Journal www.biochemj.org

© The Authors Journal compilation © 2014 Biochemical Society

A **T115**

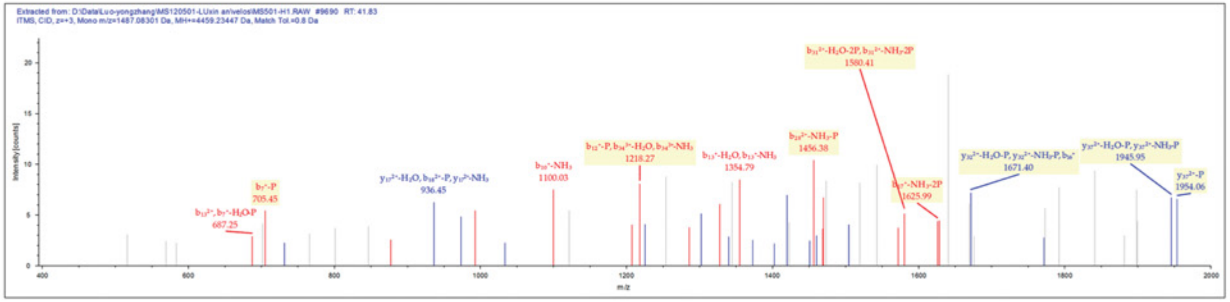

#1	b⁺	b²⁺	b³⁺	Seq.	y⁺	y²⁺	y³⁺	#2
1	88.03931	44.52329	30.01795	S				41
2	145.06078	73.03403	49.02511	G	4372.10882	2186.55805	1458.04113	40
3	326.07479	163.54103	109.36311	T-Phospho	4315.08735	2158.04731	1439.03397	39
4	454.16976	227.58852	152.06144	K	4134.07334	2067.54031	1378.69597	38
5	525.20688	263.10708	175.74048	A	4005.97837	2003.49282	1335.99764	37
6	672.27530	336.64129	224.76328	F	3934.94125	1967.97426	1312.31860	36
7	803.31580	402.16154	268.44345	M	3787.87283	1894.44005	1263.29580	35
8	932.35840	466.68284	311.45765	E	3656.83233	1828.91980	1219.61563	34
9	1003.39552	502.20140	335.13669	A	3527.78973	1764.39850	1176.60143	33
10	1116.47959	558.74343	372.83138	L	3456.75261	1728.87994	1152.92239	32
11	1244.53817	622.77272	415.51757	Q	3343.66854	1672.33791	1115.22770	31
12	1315.57529	658.29128	439.19661	A	3215.60996	1608.30862	1072.54151	30
13	1372.59676	686.80202	458.20377	G	3144.57284	1572.79006	1048.86247	29
14	1443.63388	722.32058	481.88281	A	3087.55137	1544.27932	1029.85531	28
15	1558.66083	779.83405	520.22513	D	3016.51425	1508.76076	1006.17627	27
16	1671.74490	836.37609	557.91982	I	2901.48730	1451.24729	967.83395	26
17	1838.74326	919.87527	613.58594	S-Phospho	2788.40323	1394.70525	930.13926	25
18	1969.78376	985.39552	657.26610	M	2621.40487	1311.20607	874.47314	24
19	2082.86783	1041.93755	694.96079	I	2490.36437	1245.68582	830.79297	23
20	2139.88930	1070.44829	713.96795	G	2377.28030	1189.14379	793.09828	22
21	2267.94788	1134.47758	756.65414	Q	2320.25883	1160.63305	774.09113	21
22	2415.01630	1208.01179	805.67695	F	2192.20025	1096.60376	731.40493	20
23	2472.03777	1236.52252	824.68411	G	2045.13183	1023.06955	682.38213	19
24	2571.10619	1286.05673	857.70691	V	1988.11036	994.55882	663.37497	18
25	2628.12766	1314.56747	876.71407	G	1889.04194	945.02461	630.35216	17
26	2775.19608	1388.10168	925.73688	F	1832.02047	916.51387	611.34501	16
27	2938.25940	1469.63334	980.09132	Y	1684.95205	842.97966	562.32220	15
28	3025.29143	1513.14935	1009.10199	S	1521.88873	761.44800	507.96776	14
29	3096.32855	1548.66791	1032.78103	A	1434.85670	717.93199	478.95708	13
30	3259.39187	1630.19957	1087.13547	Y	1363.81958	682.41343	455.27804	12
31	3372.47594	1686.74161	1124.83016	L	1200.75626	600.88177	400.92360	11
32	3471.54436	1736.27582	1157.85297	V	1087.67219	544.33973	363.22891	10
33	3542.58148	1771.79438	1181.53201	A	988.60377	494.80552	330.20611	9
34	3671.62408	1836.31568	1224.54621	E	917.56665	459.28696	306.52707	8
35	3799.71905	1900.36316	1267.24453	K	788.52405	394.76566	263.51287	7
36	3898.78747	1949.89737	1300.26734	V	660.42908	330.71818	220.81454	6
37	3999.83515	2000.42121	1333.94990	T	561.36066	281.18397	187.79174	5
38	4098.90357	2049.95542	1366.97271	V	460.31298	230.66013	154.10918	4
39	4211.98764	2106.49746	1404.66740	I	361.24456	181.12592	121.08637	3
40	4313.03532	2157.02130	1438.34996	T	248.16049	124.58388	83.39168	2
41				K	147.11281	74.06004	49.70912	1

B **T425**

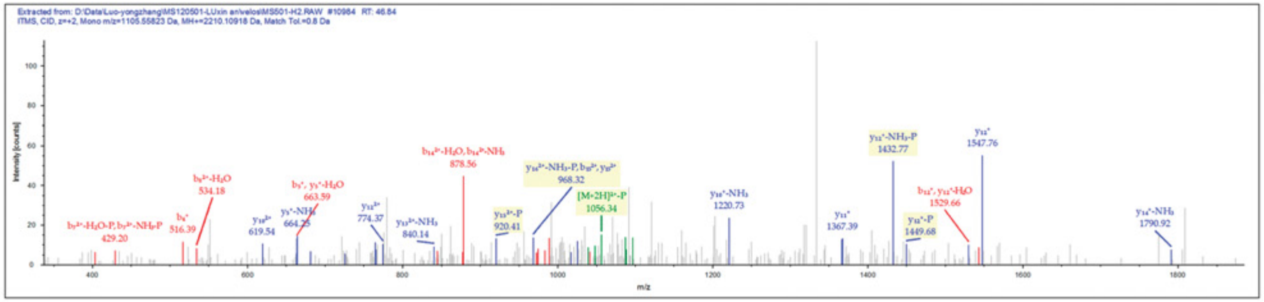

#1	b⁺	b²⁺	Seq.	y⁺	y²⁺	#2
1	161.03793	81.02260	C-Carbamidomethyl			17
2	274.12200	137.56464	L	2049.97811	1025.49269	16
3	403.16460	202.08594	E	1936.89404	968.95066	15
4	516.24867	258.62797	L	1807.85144	904.42936	14
5	663.31709	332.16218	F	1694.76737	847.88732	13
6	844.33110	422.66919	T-Phospho	1547.69895	774.35311	12
7	973.37370	487.19049	E	1366.68494	683.84611	11
8	1086.45777	543.73252	L	1237.64234	619.32481	10
9	1157.49489	579.25108	A	1124.55827	562.78277	9
10	1286.53749	643.77238	E	1053.52115	527.26421	8
11	1401.56444	701.28586	D	924.47855	462.74291	7
12	1529.65941	765.33334	K	809.45160	405.22944	6
13	1658.70201	829.85464	E	681.35663	341.18195	5
14	1772.74494	886.87611	N	552.31403	276.66065	4
15	1935.80826	968.40777	Y	438.27110	219.63919	3
16	2063.90323	1032.45525	K	275.20778	138.10753	2
17			K	147.11281	74.06004	1

C **T603**

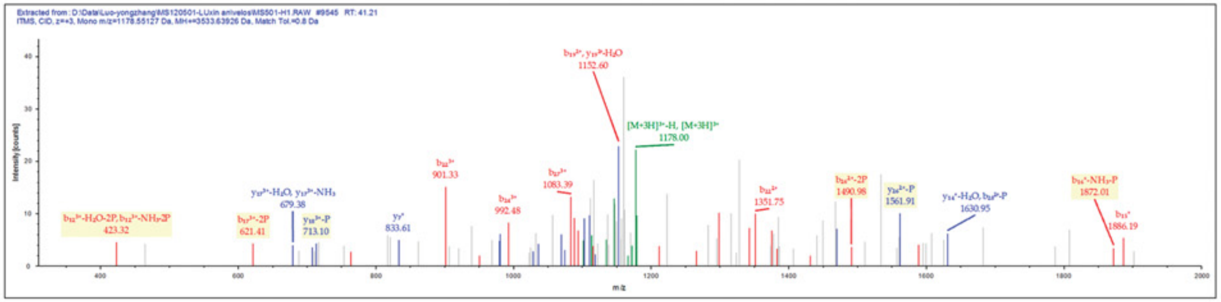

114.09135	57.54931	38.70197	L				29
213.15977	107.08352	71.72477	V	3420.50112	1710.75420	1140.83856	28
314.20745	157.60736	105.40733	T	3321.43270	1661.21999	1107.81575	27
481.20581	241.10654	161.07345	S-Phospho	3220.38502	1610.69615	1074.13319	26
578.25858	289.63293	193.42438	P	3053.38666	1527.19697	1018.46707	25
738.28923	369.64825	246.76793	C-Carbamidomethyl	2956.33389	1478.67058	986.11615	24
898.31989	449.66358	300.11148	C-Carbamidomethyl	2796.30323	1398.65525	932.77259	23
1011.40396	506.20562	337.80617	I	2636.27258	1318.63993	879.42904	22
1110.47238	555.73983	370.82898	V	2523.18851	1262.09789	841.73435	21
1211.52006	606.26367	404.51154	T	2424.12009	1212.56368	808.71155	20
1298.55209	649.77968	433.52221	S	2323.07241	1162.03984	775.02899	19
1479.56610	740.28669	493.86022	T-Phospho	2236.04038	1118.52383	746.01831	18
1642.62942	821.81835	548.21466	Y	2055.02637	1028.01682	685.68031	17
1699.65089	850.32908	567.22181	G	1891.96305	946.48516	631.32587	16
1885.73021	943.36874	629.24825	W	1834.94158	917.97443	612.31871	15
1986.77789	993.89258	662.93081	T	1648.86226	824.93477	550.29227	14
2057.81501	1029.41114	686.60985	A	1547.81458	774.41093	516.60971	13
2171.85794	1086.43261	724.62416	N	1476.77746	738.89237	492.93067	12
2302.89844	1151.95286	768.30433	M	1362.73453	681.87090	454.91636	11
2431.94104	1216.47416	811.31853	E	1231.69403	616.35065	411.23619	10
2588.04216	1294.52472	863.35224	R	1102.65143	551.82935	368.22199	9
2701.12623	1351.06675	901.04693	I	946.55031	473.77879	316.18829	8
2848.16164	1424.58446	950.05873	M-Oxidation	833.46624	417.23676	278.49360	7
2976.25661	1488.63194	992.75705	K	686.43082	343.71905	229.48179	6
3047.29373	1524.15050	1016.43609	A	558.33585	279.67156	186.78347	5
3175.35231	1588.17979	1059.12229	Q	487.29873	244.15300	163.10443	4
3246.38943	1623.69835	1082.80133	A	359.24015	180.12371	120.41823	3
3359.47350	1680.24039	1120.49602	L	288.20303	144.60515	96.73919	2
			R	175.11896	88.06312	59.04450	1

Figure S4 Determining phosphorylation sites by LC-MS

(**A**) Positive identification of the SGTKAFMEALQAGADISMIGQFGVGFYSAYLVAEKVTVITK peptide with Thr[3] and Ser[17] phosphorylation. (**B**) Positive identification of the CLELFTELAEDKENYKK peptide with Thr[6] phosphorylation. (**C**) Positive identification of the LVTSPCCIVTSTYGWTANMERIMKAQALR peptide with Ser[4] and Thr[12] phosphorylation.

© 2014 The Author(s)
The author(s) has paid for this article to be freely available under the terms of the Creative Commons Attribution Licence (CC-BY) (http://creativecommons.org/licenses/by/3.0/) which permits unrestricted use, distribution and reproduction in any medium, provided the original work is properly cited.

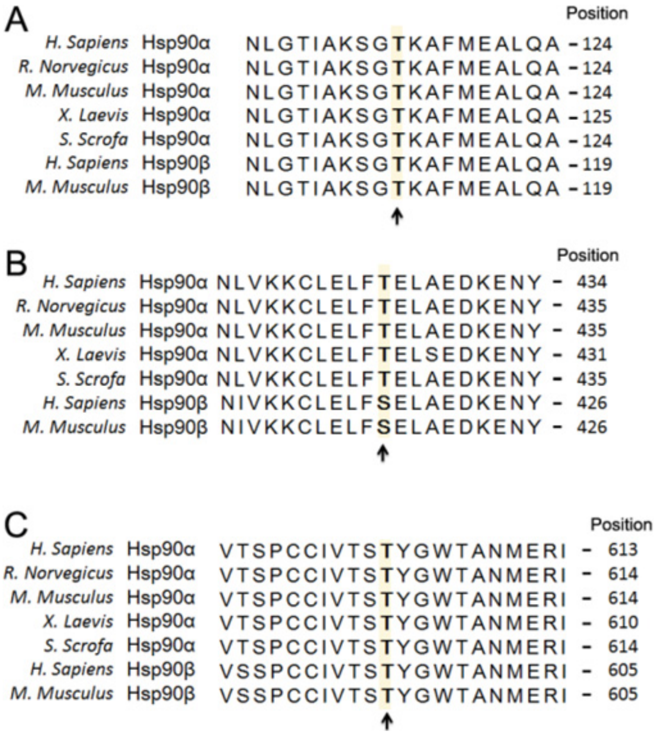

Figure S5 Diagram of the threonine set (Thr^{115}/Thr^{425}/Thr^{603}) in Hsp90α shows alignments of the sequences from different organisms and isoforms of Hsp90 and relative positions in tertiary structure

(**A**–**C**) Thr^{115}, Thr^{425} or Thr^{603} or their corresponding residues in other organisms/isoforms are shaded in grey and bold, showing that these phosphorylation residues are conserved in higher eukaryotes.

Received 22 July 2013/11 October 2013; accepted 14 October 2013
Published as BJ Immediate Publication 14 October 2013, doi:10.1042/BJ20130963

© 2014 The Author(s)
The author(s) has paid for this article to be freely available under the terms of the Creative Commons Attribution Licence (CC-BY) (http://creativecommons.org/licenses/by/3.0/) which permits unrestricted use, distribution and reproduction in any medium, provided the original work is properly cited.
